# The evolvement of trust in response to the COVID-19 pandemic among migrants in Norway

**DOI:** 10.1186/s12939-022-01747-9

**Published:** 2022-11-03

**Authors:** Raquel Herrero-Arias, Gaby Ortiz-Barreda, Elżbieta Czapka, Esperanza Diaz

**Affiliations:** 1 Department of Welfare and Participation, Western Norway University of Applied Sciences, 5063 Bergen, Norway; 2 Community Work Research Group, Department of Welfare and Participation, University of Applied Sciences, Bergen, Norway; 3 Department of Health Promotion and Development, University of Bergen, Bergen, Norway; 4 Equity in Social Welfare and Global Development Research Group, Department of Health Promotion and Development, University of Bergen, Bergen, Norway; 5 Department of Social Sciences, Institute of Sociology, University of Gdańsk, 80-309 Gdańsk, Poland; 6 Department of Nursing and Health Promotion, Oslo Metropolitan University, Oslo, Norway; 7 Department of Global Public Health and Primary Care, University of Bergen, Bergen, Norway; 8 Unit for Migration and Health, Norwegian Institute of Public Health, Oslo, Norway

**Keywords:** Migrants, Refugees, COVID-19, Trust, Qualitative research, Authorities

## Abstract

**Background:**

The COVID-19 pandemic has had profound consequences for the world’s population, particularly for vulnerable groups like migrants who face barriers to healthcare access. Trust in authorities is crucial to any crisis management strategy implemented by a government. However, trust in authorities is linked to trust in other areas of life and it evolves during a crisis. This study explores migrants’ trust in the Norwegian government’s response to the COVID-19 pandemic.

**Methods:**

We conducted semi-structured interviews from April to May 2020 with migrants from Somalia (10), Syria (15), Sri Lanka (10), Chile (10) and Poland (10) who were living in Norway. Interviews were conducted via telephone and in participants’ mother tongue. Data were analysed thematically using the systematic text condensation method.

**Results:**

Trust was established at four levels: (i) in the personal sphere, (ii) in Norwegian society in general, (iii) in the Norwegian authorities’ management of the pandemic, and (iv) in the transnational sphere. Trust was deeply rooted in relationships with individuals, groups and entities, across countries. High trust in authorities emerged in the accounts of participants who felt they were taken care of in the diverse relationships they established in Norway, particularly during the crisis.

**Conclusion:**

Pandemics create more vulnerability but also opportunities for trust-building. Trust-building can be fostered through relationships in the host country that provide the foundation for migrants to feel included. Healthcare providers are in a position from which they can nurture trust as they can build relationships with migrants over time.

**Supplementary Information:**

The online version contains supplementary material available at 10.1186/s12939-022-01747-9.

## Introduction

The COVID-19 pandemic has threatened nations’ security, prosperity and social order, posing short- and long-term health, economic and social challenges worldwide (Guan et al., 2020, p.2). To mitigate the adverse effects of the pandemic and to contain the spread of the virus, governments have implemented strict protocols. By April 2020, more than half of the world’s population had experienced strong COVID-19 containment measures in the face of increasing uncertainty and social and economic disruption. The virus and its prevention and control measures exacerbated the inequalities between and within countries since groups living in vulnerable situations suffered the worst health, social and economic consequences of the pandemic (WHO, 2020).

Persuading people to comply with COVID-19 prevention and control measures is the cornerstone on which to manage the health and humanitarian crisis that unfolded in the wake of the pandemic. In this context, trust, an essential condition for well-functioning relationships at interpersonal, societal and transnational levels (Dekker and Uslaner, 2001), plays a significant role in the success of governments’ response to the pandemic. Studies found that high trust in government and authorities is associated with positive public perceptions of the government response to COVID-19 and compliance with preventive measures (Lazarus et al., 2020; Seale et al., 2020). However, migrants often present a higher level of distrust due to their experiences of discrimination and inequality among other factors (Alesina and La Ferrara, 2002). Yet trust cannot be seen as static since it is constantly built and negotiated (Giddens, 1990).

Drawing on interviews with migrants from Poland, Chile, Syria, Somalia and Sri Lanka who are living in Norway, this study explores the issue of trust among these migrant groups in the context of the Norwegian authorities’ response to the COVID-19 pandemic. In this article, migrants are defined as persons born abroad of two foreign-born parents (Statistics Norway, 2022).

### Trust and healthcare

Also referred to as “the foundation of society” (Fukuyama, 1995), trust is “the optimistic acceptance of a vulnerable situation in which the trustor believes the trustee will care for the trustor’s interests” (Hall et al., 2001, p. 615). The notion of trust implies “a degree of vulnerability and risk” as “it is rooted in the expectation that the other will have concern for your interests” (Gilson, 2006, p. 360). We can distinguish several levels of trust, from individual relationships to trust in social systems such as institutions, professionals, authorities and society. Yet it is essential to note that both the micro and macro levels are interrelated since one-to-one relationships provide a basis for institution-based trust in strangers, which lays the foundation for general social trust (Giddens, 1990).

Trust is of crucial relevance for healthcare systems as a requisite for the acceptance of services and, therefore, the successful delivery of care and the legitimation of medical practice (Giddens, 1990). A trusting relationship between patient and healthcare provider is based on expectations and actions, and is shaped by several actors and components operating at multiple levels. For instance, managerial and organizational practices, political processes and personal relationships between patients and healthcare providers may hinder or promote trust within health systems (Gilson, 2006). In the context of health crises, trust in health systems and authorities gains importance as a requisite for compliance with policies and measures and, therefore, for the effective management of the situation (Everett et al., 2021).

Research on trust in healthcare and vaccination has shown that trust is generated and negotiated over time in relationships with multiple individuals and organizations (Charura et al., 2021; Jamison et al., 2019; Næss, 2019; Razai et al., 2021). In this context, the impact that contextual factors have on compromising or strengthening trust in healthcare has been acknowledged (Jamison et al., 2019; MacDonald, 2015). This is particularly true in research with migrants and ethnic minorities who need to navigate complex structural and personal factors when negotiating trust. Racism and discrimination, personal experiences with culturally insensitive healthcare and differences in culture and communication are examples of additional layers of complexity for migrants’ and minorities’ negotiation of trust in healthcare (Ayazi, 2006; Herrero-Arias and Diaz, 2021; Jamison et al., 2019; Næss, 2019; Razai et al., 2021; Svenberg et al., 2011). Furthermore, trust-building between migrants, institutions and the host society arises as an imperative for the integration of migrants (Haj-Younes et al., 2021).

### The COVID-19 pandemic in Norway

Norway is a country with a solid and stable economy, and a welfare state model characterized by high taxes, decommodification, social trust and promotion of equality (Esping-Andersen, 1990; Fukuyama, 1995). In addition to higher societal trust in the government (OECD, 2017), the country has a tight culture with regard to compliance with social norms (Gelfand et al., 2011).

During the pandemic, Norway experienced a lower number of infected citizens per 100,000 inhabitants than other European countries. “Competent politicians, a high-trust society with a reliable and professional bureaucracy, a strong state, a good economic situation, a big welfare state, and low population density” are factors that can explain the successful handling of the COVID-19 crisis by the Norwegian government (Christensen and Lægreid, 2020, pp. 778). The first cases of infection were registered in the country at the end of February 2020. On 12 March, the government issued the strictest measures in peacetime, including mandatory quarantine, significant restrictions on social contact and movement and border controls. Schools and workplaces were closed, and leisure and cultural activities were banned (Norwegian Government, 2020). Although the COVID-19 containment measures were strict, Norway did not experience a complete lockdown as authorities frequently appealed to people’s solidarity and trust in the government’s capacity and willingness to manage the crisis (Christensen and Lægreid, 2020; Moss and Sandbakken, 2021). Economic packages to mitigate the negative economic impact of the containment measures were also introduced. On 8 April, the government decided to ease COVID-19 restrictions gradually; an example of this was the reopening of kindergartens (20 April), primary schools (27 April) and all schools (11 May).

Trust in political and health authorities was strengthened during the pandemic (Medborgerpanelet 2020), possibly because of the communication and crisis management approach that was followed (Christensen and Lægreid, 2020). Unlike other countries where the pandemic was politicized, the Norwegian response to COVID-19 during the first few months was marked by consensus and collaboration between political and professional executives, typical of the Norwegian political decision-making process.

Another reason why Norway had lower death rates and higher intensive care survival rates is the high quality and accessibility of its healthcare services (Christensen and Lægreid, 2020). However, migrants face systemic barriers to healthcare due to language, low socio-economic status and sociocultural factors (Czapka and Sagbakken, 2016; Herrero-Arias and Diaz, 2021; Småland Goth and Berg, 2011). This issue was highlighted during the pandemic as migrants have been over-represented in rates of infections and hospitalizations (Diaz et al., 2020). Explanations for the differential impact of COVID-19 across demographic groups include crowded housing conditions, low income and type of occupation, factors that may increase migrants’ exposure to the virus and hinder them from protecting themselves and understanding health-related information (Jervelund and Eikemo, 2021; Kjøllesdal et al., 2021).

## Methodology

### Study design

This phenomenological qualitative study is part of INNCovid.Norge, a research project initiated by the University of Bergen in cooperation with other institutions in response to the COVID-19 pandemic. We interviewed 26 men and 29 women from Somalia (10), Syria (15), Sri Lanka (10), Chile (10) and Poland (10) (Table 1). Participants were recruited through key members of the migrant communities in Norway, personal networks of the researchers, advertising on existing Facebook groups used by migrants in Norway and snowball sampling.

**Table 1:** Sociodemographic characteristics of the participants

	Gender	Age category	Country of origin/ parents’ origin	Education	Period of stay in Norway (years)
1	F	41–50	Poland	university	16
2	F	41–50	Poland	high school	9
3	M	31–40	Poland	high school	10
4	M	31–40	Poland	secondary (vocational)	15
5	F	51–60	Poland	university	5
6	F	21–30	Poland	secondary (vocational)	4
7	F	41–50	Poland	university	3
8	M	51–60	Poland	university	11
9	F	61–70	Poland	university	39
10	F	31–40	Poland	university	8
11	F	51–60	Chile	secondary	16
12	M	51–60	Chile	secondary	32
13	M	30–40	Chile	secondary	11
14	F	61–70	Chile	basic	25
15	F	41–50	Chile	secondary (vocational)	32
16	M	21–30	Chile	vocational course	9
17	F	61–70	Chile	vocational course	40
18	M	31–40	Chile	master student	1
19	M	31–40	Chile	university	10
20	F	61–70	Chile	vocational course	32
21	F	61–70	Syria	high school	5
22	M	41–50	Syria	secondary	3
23	F	61–70	Syria	high school	4
24	M	41–50	Syria	primary	3
25	F	31–40	Syria	high school	6
26	F	41–50	Syria	secondary	4
27	F	21–30	Syria	high school	3
28	F	31–40	Syria	university	9
29	M	41–50	Syria	no school	2
30	M	51–60	Syria	secondary	7
31	F	41–50	Syria	high school	5
32	M	31–40	Syria	primary	1
33	M	41–50	Syria	high school	5
34	F	61–70	Syria	high school	5
35	M	41–50	Syria	secondary	5
36	F	21–30	Sri Lanka	university	Born in Norway
37	M	31–40	Sri Lanka	university	16
38	M	51–60	Sri Lanka	high school	39
39	F	31–40	Sri Lanka	university (bachelor)	15
40	M	51–60	Sri Lanka	high school	31
41	M	51–60	Sri Lanka	high school	30
42	F	51–60	Sri Lanka	high school	35
43	M	41–50	Sri Lanka	high school	10
44	M	41–50	Sri Lanka	university (bachelor)	11
45	F	80–90	Sri Lanka	No school	8
46	F	41–50	Somalia	primary school	22
47	F	31–40	Somalia	primary school	12
48	F	41–50	Somalia	university	31
49	M	41–50	Somalia	high school	13
50	F	21–30	Somalia	high school	10
51	M	41–50	Somalia	secondary school	17
52	M	51–60	Somalia	high school	32
53	M	51–60	Somalia	University (bachelor)	30
54	M	41–50	Somalia	university (bachelor)	6
55	F	51–60	Somalia	high school	25

### Data collection

Data were collected using semi-structured interviews (Additional file 1). All participants consented to being interviewed for the study and were offered a 150 NOK gift card in appreciation of their time. Participants were also asked to provide socio-demographic information (age, sex, education level, length of stay in Norway, and civil status). Interviews were conducted from April to May 2020 by bilingual researchers in participants’ mother tongue. The interviews, which lasted 30 to 45 min each, were conducted over the telephone because of the COVID-19 containment measures that were in force at the beginning of the pandemic.

### Data analysis

All interview recordings were transcribed verbatim in participants’ mother tongue with one of the study researchers. The interviews were then translated into Norwegian by professional interpreters with experience in qualitative studies. Researchers read each other’s interviews in pairs. After that, ED and EC read all the transcripts from the specified languages as quality control. We used the systematic text condensation (STC) proposed by Malterud (2012) for a thematic analysis of data. The analysis process consisted of the following steps:

1) Total impression: From chaos to themes.

2) Identifying and sorting meaning units: From themes to codes.

3) Condensation: From code to meaning.

4) Synthesizing: From condensation to descriptions and concepts.

In this process, we followed an iterative approach where the codes and first themes emerged from the data and were later analysed in light of the literature. The coding followed a latent approach as code formation went beyond the descriptive or semantic content of the data to identifying the underlying assumptions.

Once the analysis was performed, the patterns of codes were identified and mapped to look for potential themes and subthemes. All the reports of the analysis were shared and discussed with the research team, and a consensus on codebooks and themes was reached. Given that STC offers a pragmatic and systematic approach, transparency, intersubjectivity, feasibility and reflexivity of the study were safeguarded. With regard to reflexivity, the researchers could possibly have an “insider” position because of their shared ethnic membership with the participants. Others can be seen as “insiders by proxy” (Carling et al. 2014) because they were migrant researchers from a different country than that of the participants. These positions promoted a comfortable atmosphere in which informants could share critical views about the host country, assuming that the interviewers would not feel offended (Carling et al. 2014). However, this could have led to bias. To minimize the influence of assumptions and subjectivities, self-reflexivity and peer-debriefing were crucial.

### Ethical considerations

Ethics applications were approved by the Regional Ethical Committee (REK) at the University of Bergen in Norway (Project number: 132585). Participants were provided with written and verbal information in their mother tongue about the aims and design of the study. Pseudonyms were used to ensure anonymity and confidentiality.

## Results

Despite not being addressed directly through questions, the issue of trust came up in all the interviews as something the participants negotiated at different levels in their lives. We identified four levels of trust negotiations and presented these thematically: in the personal sphere, in Norwegian society, in Norwegian authorities’ management of the pandemic, and in the transnational sphere. These four levels were found across all groups of informants with some nuances that we present next.

### Personal sphere

The informants felt that their response to the recommendations during the COVID-19 pandemic was a matter of personal choice. At the same time, they acknowledged that their decisions were influenced by the authorities and other people’s views and recommendations. With regard to the latter, some informants felt that high trust in family and friends could sometimes lead to not following some recommendations.

*I follow most of the measures, but there are some that I either don’t follow on purpose or I forget them…. It also happens that I meet people in private, that is, I meet friends and colleagues that I trust.* (Informant 4)

For many, the measures were perceived as a threat of loss of personal freedom.

*Terrible changes were implemented in society, and it’s in a way a restriction of personal freedom, the things we all must get used to, to starting living again…there is an interference in one’s life, interference in security, in my opinion, it’s a great interference.* (Informant 3)

Informants argued that one should trust oneself and critically assess the available information on the virus. At the core of these everyday decisions, many talked about personal values like helping others or being a role model as necessary.

*What I think is that everyone must think for himself/herself… whether the authorities say it or someone else says it, right? This is something I must think about so I don’t get it [the virus]. That’s how everyone should think.* (Informant 39)

*Honestly, it´s the innermost persuasion that is most important. The most important thing is to think about yourself, to be worried about your family or about others around you and those you love that you might infect them [because] this means that you have an inner motivation that makes you follow the measures.* (Informant 25)

Across some interviews, especially among Muslim informants, faith and religion emerged as important resources that helped informants cope with the COVID-19 crisis and the containment measures.

*I stay clean, I do wudu; those who do wudu have an advantage.* (Informant 29)

Despite religious beliefs relating to fate, informants stressed the importance of following preventive measures and being cautious.

*The risk is there, and we trust Allah, that is the first thing. But this is a pandemic, one should be careful, so one is afraid because there is a virus and we have no cure or vaccine.* (Informant 22)

*As I said earlier, “protection is better than the treatment itself”; one should be careful and take care of more things, even if there are some people who have other opinions and say, “It’s okay, one must live normally”, and “Despite what happens, only what Allah has predestined happens”. This is 100 per cent correct, but you must also take precautions; it isn’t wrong to be more careful with everything, even with those closest to you.* (Informant 25)

However, according to our informants, religion could also lead some individuals to believe that the situation was out of their control.

*It is difficult for Somalis to keep their distance, they have a habit of gathering… The most difficult thing for our people in our country is to keep our distance. And when you ask them what they are doing, they [instead] ask you: " What are you scared of? Everything is predetermined by God”.* (Informant 48)

Many participants had previously experienced crises like war, dictatorship and public health disasters. For some, these experiences created feelings of familiarity in the face of the pandemic.

*When we were in Syria, there was a spread of cholera…, same story, some died and some survived… we got through it, but it was called cholera and now it´s called corona. (Informant 21)*

However, most participants emphasized the fact that the COVID-19 pandemic was utterly different and previous experiences could not help them in coping with it.

*We experienced something similar in Chile, the dictatorship… during the daytime you could go out to shop…[but] you had to be home by 8 o’clock [in the evening], and you had to be locked up at home until 6 o’clock [in the morning]. You could go to work, you didn’t have freedom of speech and things like that, but this [pandemic] is much more than a dictatorship. This suppresses everything that is social, it suppresses you as a person because you are not used to it.* (Informant, 12)

*When there was a shortage of food, I was reminded of life in my homeland during the war. We didn’t have it so tough even during the war, but now we must go through something like this…. When it came to firing artillery shells, we [would] go into bunkers and be safe when the plane came. But now, we are afraid to touch someone who is next to us. It’s stressful.* (Informant 37)

For other informants, the pandemic was a new threat to still existing wounds.

*It’s just like with the Syrian war; from 2011 to 2015 they said, “Now, it’s soon finished”, “It will be finished tomorrow”, but it never finished and it is still going on. So, it is psychologically challenging, and we can no longer cope with the mental stress. We have already been destroyed from before we left our country; we cannot cope with more.* (Informant 33)

*This situation has also evoked painful memories: the war in the homeland, insecurity and isolation.* (Informant 49)

Participants’ previous experiences with crises and authorities’ emergency management could also lead to scepticism and distrust in the COVID-19 response. For instance, Polish informants talked about the Chernobyl accident that, from their viewpoint, was not handled in a trustworthy manner.

*I can associate it with the Chernobyl accident… I remember from my childhood that we were not allowed to be home, but I remember that there was enormous panic and one had to drink iodine… I read yesterday that Oslo Municipality has already notified that in kindergartens and schools there will be—if there is any pollution—distribution of iodine tablets… we have already received such information…so I have to get iodine, because if something happens and people start buying up [iodine] at pharmacies there may be a shortage.* (Informant 2)

Among Polish informants, we also found conspiracy theories to explain the current global crisis.

*What I say to myself is that it [COVID-19] must be a kind of biological warfare because there are so many infected and so many died from this …I think that the countries that have large numbers of older people would get rid of the older society so that they don’t have to pay pensions. Maybe I should not say more*. (Informant 2)

### Living in Norwegian society

Some informants talked about their Norwegian neighbours as distant subjects with whom they had zero to very little interaction before the pandemic. However, in instances when good personal relations with Norwegian neighbours were previously established, these relations were viewed as a valuable source of social support to help cope against isolation. With neighbours it was possible to maintain social distance and still have some social interaction.

*As I said, my grandchildren have no contact with me because my daughters don’t let them. But I talk to my neighbours; I help a neighbour who lives above me because she has problems and I talk to her, we talk at a 1m distance … [it’s] the same with people on the street, we talk at a distance, but we talk, that is, the contact is the same, but at a distance…there is no physical proximity as before.* (Informant 17)

Across the data, workplaces could be positively or negatively related to trust during the pandemic. For instance, workplaces could be seen as reliable and useful sources of information and care, which promoted trust in employers who were seen as caring and competent. This also created trust among migrants who were second-line leaders and would act as role models for their working team, even when they were personally sceptical to the recommendations. An example is the next quote from a Chilean informant, who did not think that the measures were the most effective solution to the pandemic. However, because of his position at work, he followed the recommendations and informed his employees about them.

*We still read the news because we must be informed; unfortunately, we are responsible for kindergartens and schools and so we must know and inform employees about the latest news. My boss sent us an email about how the pandemic is affecting people, how it’s being handled…everyday, my boss has meetings with other managers and he is asked how things are going, how people are doing; so, we have to send him information and also constantly speak with the employees; we must be informed, unfortunately.* (Informant 12)

However, there were also several examples of work that were disempowering and a hindrance to taking precautions, especially among essential workers. These types of work created less trust in the employer, the authorities and Norwegian society in a more general way.

At the time of the interviews, schools were about to reopen, creating scepticism primarily because schools remained closed in other countries. Most informants hoped everything would go well; they did not have any other choice than to trust the new measures. However, a special case was reported by a Chilean single mother who had two children, one of whom was in need of special educational support. The help and support she got from the schoolteacher through the lockdown prompted her trust in the school’s capacity to manage the reopening safely.

*They made me go [to the school] to get the books and the teacher said to me… “Take it step by step with him because it’s more difficult for him. So, take it step by step”, and this made me so happy because it means that the state also cares about my children. Even though they were at home, and they knew they were fine at home…they still cared about learning….You appreciate someone caring about you and your children.*

*My girl has asthma…I asked her teacher to find out if I could send her to school. She had the patience to explain to me that children with asthma could go to school and that there was no problem with this….So, on Monday, I sent them with a face mask.* (Informant 15)

Most informants reported an ambivalent view of Norwegian society with regard to how it responded to the pandemic. On the one hand, Norwegian society was often described as a society with a “tight” culture, where people commit to the rules and take responsibility for their actions, and as a well-prepared society with a high level of welfare, all of which were generally seen as positives with which to fight the pandemic. Informants who had positive views of the host society and the healthcare system showed more trust in the authorities in general.

*Norway can be said to have already passed the danger zone because Norwegian society listens to the messages they receive, they don’t protest if someone gives notice…it’s a world crisis, many countries [are] struggling with the pandemic.* (Informant 34)

On the other hand, Norwegians were criticized for forgetting the rules in fine weather, when they would congregate outdoors, and for being uncritical of the information that was given out. This understanding of Norwegians as uncritically adherent to recommendations could lead to a general lack of trust in recommendations.

*If it’s a sunny day, people gather in the parks. Young people come to the parks and drink.* (Informant 40)

*One shouldn´t absorb every single piece of information uncritically. There is a lack of criticism and proper reception of information, even from the most reliable means of communication (…) Norwegians trust authorities completely uncritically*(Informant 1).

For instance, some informants felt that COVID-19 had speeded up their efforts to integrate into the host society as the pandemic required them to keep themselves informed about the situation and the measures being implemented constantly. Others shared that the pandemic had reinforced their feeling of being outsiders due to experiences of racism/discrimination.

*I have been at the supermarkets and people turn around and look at you because they are afraid of you, [thinking] “This man has the virus”…I think that this [pandemic] is affecting people in a very strong psychological way. Everyone, especially in this country where everyone is half classist and [half] racist…[is] going to see you as a weirdo, as a carrier, [as] “this dark-skinned man”.* (Informant 12)

Participants also mentioned that the media presented immigrants’ shopping centres as hotspots for contagion and used “culture” to explain the over-representation of migrants in the infection rates. To respond to this, many informants pointed out that Norwegians also have their weaknesses when it comes to compliance, for example, gathering in the park when the weather was good.

### Norwegian authorities’ management of the pandemic

Most respondents stated that the available information on COVID-19 in Norway generally elicited feelings of empowerment and trust. However, this information was unavailable to those with low Norwegian and digital competence.

*The authorities were late [in] informing minority populations. The authorities were delayed in using the correct channels and didn’t translate the information into several languages.* (Informant 51)

*The measures didn´t reach everyone quickly enough. The authorities could have (…) made plaques with understandable language and translated them into different languages and which could be put at places where migrants meet.* (Informant 48)

While transparency in the disagreements between health authorities prompted trust among some informants, the perceived internal conflicts between the various health authorities created mistrust for others. Likewise, we found people criticizing the lack of evidence to support the constant changes and lack of questioning on the longer-term consequences, especially from an economic point of view.

*There is doubt whether the authorities give us trustworthy information. Besides, they say that you shouldn’t use a face mask, the WHO also said that you don’t need to use it…. They said that ordinary healthy people didn’t need to use it. And now the WHO says that it can be used in some places. And that’s what the Norwegian authorities follow.… One gets a feeling that they are constantly changing the messages they give.… they often change their minds.* (Informant 37)

*I was surprised by the measures… there are major consequences of such actions, especially for the economy. I know how many people lost their jobs … [but the] consequences can be much worse. We are talking about suicide, depression; one must be prepared for and react strongly… to this [the restrictions] more than to the virus.* (Informant 6)

Furthermore, past experiences with the Norwegian health system also influenced whether informants trusted how the health crisis was handled. For instance, many informants trusted doctors with a migrant background. We also found a preference for continuity of care, that is, consultations with their own general practitioners (GPs) over emergency rooms.

*If I got the virus, I would go to my doctor. I have great confidence in him … I have no confidence in the emergency room.* (Informant 17)

*I would rather go to my GP because the GP can tell you: “No, it´s only a cold”, but in the emergency room, they don’t know you and so they can just say that you actually have [the] virus.* (Informant 19)

To cope with conflicting information in the context of the pandemic, informants relied on the cultural health capital of their “own” healthcare professionals in Norway, many of whom started making information videos in the community language. Cultural or religious groups and non-governmental organizations (NGOs) played a significant role in helping spread information on COVID-19 and its control and prevention measures to immigrant groups. Some participants had a positive view of the collaboration with the municipalities and affirmed that these organizations had taken responsibility to disseminate adequate information and had helped reach people who could be difficult to get otherwise. However, some reflected upon the inequalities that approach could pose, as not all immigrant groups were well organized. Several informants had themselves experienced shouldering too much responsibility as key persons in the community without having any official role. This could sometimes lead to conflict with family and friends, and create feelings of hopelessness and lack of trust in the authorities who did not take on the role themselves.

*There are many who contact me and think I know everything. It’s as though because I work in the district, I’m a resource for the district. People call me round the clock: “What are we going to do now?“…. We aren’t responsible for others. I’ve called and reassured many families. They call me and ask what they should do. Some mothers felt hopeless and complained about their spouses working in occupations that made them vulnerable to COVID-19. They are asking for measures from the authorities to force fathers to stay at home so that they don’t bring the virus home. This is especially true in those cases where the man got infected in the workplace and infected the rest of the family, and didn’t receive external help. What should I say when a neighbour asks for my help? I had back pain at the time and said I couldn’t shop for them. This isn’t easy.* (Informant 55)

Yet it is important to note that most informants generally trusted the manner in which Norwegian authorities managed the crisis despite the uncertainty of the situation. Respondents talked about the respect, gratitude and pride they had for the Norwegian authorities. They reflected on the country’s security, the information relating to COVID-19 shared with the people, and the advantage of having a solid economy and low population. They also appreciated the authorities’ proportional response to the threat of COVID-19 in an efficient way. Some named the prime minister herself as a source of trust.

*Well, in general, we are privileged in Norway. The main reason for this is that there is a very small population. The country is prosperous. In any case, citizens [in Norway] get better help than what happens in other countries. We should be grateful for such care.* (Informant 9)

### Transnational sphere

In nearly all the interviews, the connection the informant had to at least two transnational realities was clearly evident. Many had contact with family members living abroad, and some were responsible for the health of those back in their home country. These informants would share the Norwegian authorities’ recommendations with their relatives.

*So, I had to talk with my grandma. She likes to go out and do things, although she has hip problems….She has been isolated for more than a month, unable to go out. One day she called me and told me she was going to go out… I said, “No, you cannot because you would expose yourself a lot, you are at risk, we don’t know what can happen”.* (Informant 16)

Most participants searched for information on the COVID-19 response in different countries and compared the situation and the recommendations with what was going on in Norway. Many thought the situation in Norway was much better compared to most countries because of its welfare system and wealth, comprehensive healthcare system and less politicized decision-making. Others also reflected on the “mild” lockdown in Norway compared to other countries, and how it had a lesser impact on the mental health of Norwegians. Moreover, we found participants’ concern with regard to COVID-19 to extend beyond their home countries to other developing countries with fewer chances to fight the pandemic. As one informant stated, “Previous experiences make you think more globally”.

## Discussion

We explored migrants’ trust in Norwegian institutions and authorities’ response to COVID-19 during the first months of the pandemic. Our findings highlight the interaction between factors at multiple levels (personal, relational, societal and institutional) facilitating or hindering trust and compliance with prevention measures during the pandemic. While the interplay across multiple levels of analysis is previously outlined in the biopsychosocio-ecological model (Borrel-Carrió et al., 2004), our findings suggest that this interaction is more complex in migration contexts since it intersects with past and present experiences in both host and origin countries. Moreover, as a crisis that unleashed disruption in migrants’ lives, the pandemic itself was a factor that could either serve as a barrier or facilitator to building trust in authorities, institutions and the host society.

We claim that personal values, beliefs and religion can influence attitudes and perceptions towards risk and, consequently, judgments about how the pandemic was managed. On the one hand, values of solidarity for vulnerable groups and unity promoted a sense of community that provided a foundation for trust and compliance with COVID-19 measures. On the other hand, values of individuality and freedom elicited criticism towards the measures and distrust in the handling of the pandemic because its success depended on compliance from others. As Van Bavel et al. discussed (2020, p. 464), finding a balance between freedom and constraint is important during the management of a pandemic. In this aspect, we argue that informants negotiated individualistic and collectivist values and tightness and looseness in cultural norms at the personal level. This balancing act was shaped but not determined by cultural background and life experiences. For instance, some informants stated that they come from cultural contexts where social norms are more permissive. Yet within this group we found different interpretations of the COVID-19 prevention measures implemented and the levels of compliance. While some participants pointed to cultural looseness as a challenge that made it difficult for their countries of origin to handle the pandemic, others emphasized freedom against restrictions and fear.

Religion was given as a factor that promoted trust, especially among Muslim participants. For some informants, religion provided the foundation for embracing compliance with measures for a person to become a role model during a crisis and to protect vulnerable groups. Furthermore, despite religious beliefs relating to fate, we found that participants emphasized the importance of being cautious. Interestingly, they also referred to third persons who believed that their health and future were in “God’s hands” and, consequently, health authorities’ recommendations would not necessarily guarantee them protection against COVID-19. The previous examples illustrate the great range of variation in individuals’ meaning-making to cope with an uncertain situation all of them were also facing with a relatively similar religious point of view (Sandbakken and Moss, 2021).

The frontiers between trust and compliance for other reasons like social control and avoidance of sanctions have been discussed in the literature (Gilson, 2003), and should be studied further. Drawing on informants’ experiences and reflections, we discuss that their relationships with other people in Norwegian society can greatly influence their compliance with COVID-19 prevention measures and trust in authorities’ response to the pandemic. For instance, a few participants felt safe among family and friends who were not seen as a threat to their health even though they could be a source of exposure. In these cases, trusting the behaviour of those closest to them could lead to non-compliance with measures such as social distancing.

At this relationship level, transnationalism had an impact on migrants’ perceptions. Participants grappled with the different realities of managing the pandemic through their relationships with relatives and friends in their countries of origin. This led them to compare countries’ responses to COVID-19, which would then reinforce or diminish their trust in the Norwegian authorities. For some, the COVID-19 management approach adopted in Norway was too soft compared to that of their countries of origin. In this context, participants would follow measures that had not been implemented in Norway yet to protect themselves and their families (e.g. wearing face masks). This could, in turn, be misunderstood by the host society as a sign of infection which could then turn into stigma. Others expressed high trust in the Norwegian authorities compared to their countries of origin that faced greater challenges in responding to the pandemic successfully. This created concern for the health of relatives back in their home countries, with participants sharing the recommendations made by the Norwegian authorities. In the accounts of this group, we found more appreciation and trust in the Norwegian government’s COVID-19 response, which can be seen as a coping strategy aimed at reducing negative emotions and promoting positive reinterpretations of their situation (Sandbakken and Moss, 2021).

Notably, the relationships forged in Norway emerged as a key contributing factor for trust-building because these provided the foundation for a feeling of inclusion in the host country where the participants were facing the health crisis. Fostering belonging to a shared social identity facilitates crisis management (Ellemers et al., 2002; Haslam and Reicher, 2006). In our data, trust in authorities particularly emerged in the accounts of participants who felt they were taken care of in a variety of relationships in the host country (e.g. with employers and neighbours). This shows how the micro and macro levels of trust are interrelated (Giddens, 1990) as trust in authorities and strangers was grounded in interpersonal relationships characterized by recognition and care. These interpersonal experiences made our informants feel included and safe and, therefore, prompted their trust in the authorities’ intentions. These findings support studies that found that experiences in host countries are more important for social trust than the culture of origin (Dinesen, 2012).

Past and present relationships with healthcare providers provided the foundation for a feeling of being taken care of, which was a significant source of trust in the Norwegian authorities. Unfamiliarity with the Norwegian healthcare system and negative experiences with healthcare provision attributed to the migrant background (language barrier, lack of cultural competence) could translate into scepticism towards healthcare providers, leading to distrust (Charura et al., 2021). On the contrary, informants who had over the years built good relationships with their GPs based on communication and recognition trusted this healthcare provider for information and care during the pandemic. Based on our findings and in line with other studies (Haj-Younes et al., 2021; Latkin et al., 2021; Razai et al., 2021), we argue that healthcare providers like GPs are in a position to foster trust-building as they have the opportunity to build relationships with minority groups over time.

Perceived discrimination shapes migrants’ distrust and willingness to engage with the host society (Alesina and La Ferrara, 2002; Horenczyk et al., 2013; Van Bavel et al., 2020). This issue came up in our study because the feeling of being an outsider prompted distrust in the handling of the pandemic. It is relevant to note here that the pandemic also shaped experiences of discrimination or inclusion. This is consistent with research showing that pandemics reinforce racism and xenophobia (Devakumar et al., 2020).

Perception of the government’s capacity to handle the crisis also influences individual behaviours (Brandsen and Honingh, 2016). In our study, participants positively assessed the Norwegian authorities’ capacity to successfully manage the pandemic and this, in turn, reinforced their trust. However, a comparison with their home countries, often with fewer resources than Norway but implementing several measures, could lead to criticism of Norway as a country that could have had put into action more measures given its wealth and welfare system.

Norwegians were generally perceived as compliant with regard to norms and Norwegian culture was viewed as “tight”, meaning authorities could rely on citizens’ adherence to the rules and norms. This issue was discussed across interviews. For some informants, cultural tightness reinforced their trust in other’s behaviour and compliance with measures and, in turn, in the government response to the pandemic, which was dependent on citizens’ behaviour. However, others associated the tight Norwegian culture with a lack of critical reflection on the management of the crisis, which led to distrust. This is in line with research conducted among Norwegian citizens (Medborgerpanelet, 2020) which found out that while trust in authorities increased during the pandemic, trust among citizens decreased. This is probably related to the context of social isolation and a health crisis whose successful management depends on the cooperation of the entire population (Christensen and Lægreid, 2020).

Political polarization and politicization create distrust during a crisis, which many countries experienced during COVID-19 (Hart et al., 2020; Latkin et al., 2021). In Norway, the consensus-based approach between political and administrative executives (Christensen and Lægreid, 2020) and the narrative used by the government calling for collective action (Moss and Sandbakken, 2021) promoted collaboration rather than polarization and isolation. However, some of our informants did not always experience the government’s communication strategy in these terms. First, it is important to note that low proficiency in the Norwegian language for some participants was a major barrier to accessing recommendations when we conducted the interviews; at the time, information relating to COVID-19 had been translated into several languages but not yet distributed. Second, negative personal experiences of informants, who felt they were outsiders in Norwegian society, may have been reinforced by societal and media discourses that blamed migrants for the import and rapid spread of COVID-19, contributing to the stigma and misinformation (Diaz et al., 2021). Based on our study, we suggest that information on measures and recommendations translated into foreign languages must be distributed through accessible platforms at the early stages of a health crisis. Our findings also call for a national strategy on migrant health that includes measures to address minority discrimination.

Transparency in the government’s communication strategy, as politicians openly disagreed over the response to the pandemic, can be considered a factor in promoting trust (Christensen and Lægreid, 2020; Moss and Sandbakken, 2021). Yet, for some of our informants, disagreements between different actors (Norwegian Institute for Public Health, government, scientific community, WHO) created distrust as the arguments were interpreted as showing lack of competence and knowledge. This may well be because of participants’ perceptions of professional competence based on their experiences with healthcare providers in their countries of origin who exercise professional authority (Herrero-Arias and Diaz, 2021).

Trust is high in contexts where individuals are willing to believe expert opinions and recommendations (Brown, 2009). It is, therefore, essential to look at the global discourses influencing informants’ attitudes and perceptions. There is a rising distrust of expert culture, conspiracy theories and misinformation being spread on social media around the globe (Dube et al., 2015; Larson et al., 2014; Yaqub et al., 2014). These are factors that diminish trust in public health infrastructure and the management of the pandemic by governments and professionals. Moreover, groups that experience discrimination, like migrants, are more likely to be sceptical about public health information and more likely to be receptive to fake news (Van Bavel et al., 2020). We found more conspiracy beliefs among Polish informants. As other authors have pointed out (Isański, 2015), this may be explained by the country’s post-communist past and, therefore, higher distrust in institutions.

## Conclusion

The evolvement of trust for immigrants during the COVID-19 pandemic was a complex relational process embedded across nations and developing at the speed of a virus. Our findings highlight that trust in authorities is rooted in interpersonal relationships and closely interrelated with feelings of integration in the host country. At the same time, our study calls for the need to consider the relevant role that life trajectory and transnationalism play in shaping individuals’ perceptions of and trust in the management of the pandemic. We claim that trust in the host country’s crisis handling is not solely influenced by culture and pre-migration experiences, but the interpersonal relationships migrants engaged in within Norway decisively shaped their trust in the authorities. Therefore, our study suggests that trust-building can be nurtured through relationships in the host country which provide the foundation for feelings of inclusion and being taken care of. Pandemics create social and economic disruption and add to the vulnerabilities that migrants experience. It is in this context that migrants negotiate their expectations of others and build trust in their motivations and actions. A better understanding of trust is paramount to increasing compliance with COVID-19 control and prevention measures among this population.

## Electronic supplementary material

Below is the link to the electronic supplementary material.

Supplementary Material 1. Interview Guide

## Data Availability

We include the interview guide as an Additional File. The dataset is not publicly available because the participants did not consent to the data being made available to other researchers. However, additional qualitative data analysis from our dataset are being published elsewhere.

## Declarations

### Authors’ information

RHA is an Associate Professor at the Department of Welfare and Participation, Western Norway University of Applied Sciences. GOB is an Associate Professor at the Department of Health Promotion and Development, University of Bergen. EC is an Assistant Professor at the University of Gdansk and an Associate Professor at the Oslo Metropolitan University. ED is a Professor at the Department of Global Public Health and Primary Care, and the Director of the Pandemic Center, University of Bergen.
