# Supplementary material for: The evolvement of trust in response to the COVID-19 pandemic among migrants in Norway
Source: Int J Equity Health. 2022 Nov 3;21:154. doi: 10.1186/s12939-022-01747-9 (PMC9632581; doi:10.1186/s12939-022-01747-9)
Supplement: Supplementary file 1 — Supplementary Material 1. Interview Guide [file 12939_2022_1747_MOESM1_ESM.docx]

**Additional file 1- Interview Guide**

1. Have you received information about the COVID-19 pandemic?

2. Where do you find information about COVID-19? How do you stay informed?

3. If you got the virus, what risk do you think it would pose

(a) to your own health?

b) to the health of your family- friends- loved ones who live in Norway and abroad?

c) to the health of everyone living in Norway?

4. What do you know about the measures and recommendations implemented by the authorities in Norway?

5. What do you think about the measures and recommendations? Do you consider them to be efficient to prevent the spread of the virus?

6. Do you consider that the measures and recommendations implemented can improve

a) your own health?

b) the health of your family and friends?

c) the health of everyone living in Norway?

7. To what extent do you and those close to you follow the measures that have been put in place, such as handwashing and limiting social contact?

8. How do you put into practice the recommendations of the authorities in your daily life?

9. If the measures lasted for a long time. What do you think might help you follow them over time?

10. Do you or someone close to you consider that there are some measures that are difficult to follow? Why? What would make compliance easier?

11. What would you do if you or someone in your family had symptoms of COVID-19? Would you inform your doctor or the emergency services so that the authorities know about it? Why?

12. Is there anything about the current situation that concerns you? If so, can you say something about it?

13. Is there anything about this situation that reminds you of past experiences? If so, can you say something about it? have you experienced something similar?

14. Norwegians often speak of "dugnad" regarding these measures. What do you think of the use of this term in this context?

15. Is there anything else related to the COVID-19 pandemic that you would like to share with us?
